# Molecular Characterization of Circulating Respiratory Syncytial Virus (RSV) Genotypes in Gilgit Baltistan Province of Pakistan during 2011-2012 Winter Season

**DOI:** 10.1371/journal.pone.0074018

**Published:** 2013-09-13

**Authors:** Uzma Bashir Aamir, Muhammad Masroor Alam, Hajra Sadia, Syed Sohail Zahoor Zaidi, Birjees Mazher Kazi

**Affiliations:** 1 Department of Virology, National Institute of Health, Chak Shahzad, Park Road, Islamabad, Pakistan; 2 Atta-ur-Rehman School of Applied BioSciences (ASAB), National University of Science & Technology (NUST), Islamabad, Pakistan; Beijing Institute of Microbiology and Epidemiology, China

## Abstract

Respiratory syncytial virus (RSV) is the major cause of acute lower respiratory tract infections in young children, but very little is known about its epidemiology and circulating genotypes in Pakistan. This study analyzed the epidemiological and molecular characteristics of RSV genotypes detected in Pakistani children less than 2 years of age with acute respiratory tract infections (ARIs) in a tertiary care hospital in Gilgit Baltistan (GB) province during 2011-12 winter season. RSV was detected in 75 out of 105 children presenting with acute respiratory infection. Male infants between 2-6 months age made up the highest percentage of RSV positive cases. Epidemiological factors such as pre-maturity, mean weight, clinical features and diagnosis when compared between RSV positive and negative groups were found to be statistically insignificant. Phylogenetic analysis classified all 75 of the RSV strains into 71 strains of subgroups A and 4 strains of subgroup B, respectively. Strains belonging to subgroups A and B were further subdivided into NA1/GA2 and BA, respectively. The nucleotide and deduced amino acid sequence identities were relatively high among these strains (>90%). Both RSV-A and RSV-B isolates had two potential N-glycosylation sites in HVR2 of G protein and with heavy O-glycosylation of serine and threonine residues (G scores of 0.5-0.7). This report highlights the significance of RSV as a dominant viral etiologic agent of pediatric ARIs, and need for continued molecular epidemiological surveys for early detection of prevalent strains and newly emerging genotypes to understand epidemiology of RSV infections in various regions of Pakistan.

## Introduction

Human respiratory syncytial virus (RSV) is a member of the *Pneumovirus* genus of the family *Paramyxoviridae* which causes annual outbreaks worldwide [1-4]. Infections with RSV may result in mild to severe illness with lower respiratory involvement leading to bronchiolitis and pneumonia. RSV represents a substantial amount of the burden of acute respiratory tract illness, particularly in the first year of life [5]. It is still the leading cause of hospitalization of children aged below 5 years in industrialized countries, and pneumonia associated mortality among children in developing countries as well [6-9]. In developing countries there are a few population-based estimates of the incidence of RSV disease. Data from a small number of studies has placed the estimated incidence of RSV-associated Lower Respiratory Infections (LRI) at around 97 to 180 episodes per 1000 child-years which are crude estimates, at best. However, these estimated rates for developing countries are 2.6 to 4.8 times the rate of RSV-associated LRI seen in the USA [10].

In temperate climate zones, RSV infections occur mainly during the winter season as epidemics that may last for up to 5 months but 40% of infections are detected during one peak month of either December or January [11-15]. Even though RSV has been recognized as an important pathogen, no effective vaccine prophylaxis and/or antiviral treatment is currently available against RSV infections [6,16].

RSV has a single-stranded, negative-sense, non-segmented RNA genome that is 15.2kb long, and encodes the genetic information for ten proteins [17-19]. The two most immunogenic RSV proteins are the Fusion (F) and Glycoprotein (G) that are expressed on the virion surface, responsible for inducing production of neutralizing antibodies [1,7,16-18]. Both within and between the major RSV subgroups, the G protein is the most variable viral protein with a minimally conserved ectodomain that contains 2 hypervariable regions (HVRs). The second HVR (HVR2) carries the C terminus of the protein and is commonly sequenced to examine the genetic variability of RSV strains within a given population [20-22].

RSV has been classified into two antigenic subgroups A and B (RSV-A and RSV-B) respectively, initially on the basis of the reactivity of the virus with monoclonal antibodies directed against the attachment glycoprotein (G protein) [1-3,17,19,23-25] and currently through genetic analyses [4,11,18,21,26-28]. The subgroups have been further subdivided into various genotypes. To date, 11 RSV-A (ON1, GA1–GA7, SAA1, NA1, and NA2) and 17 RSV-B (GB1–GB4, SAB1-SAB3, and BA1–BA10) genotypes have been identified [12].

Gilgit Baltistan (GB), formerly known as the Northern Areas, is the remotest north region of Pakistan, bordering Xinjiang province of China. This region boasts some of the highest mountain peaks and therefore has probably the most severe winter conditions in the country. Combined with remoteness of populace, and paucity of available health care, information on the epidemiology of various diseases is limited. District Headquarters (DHQ) Hospital is a 250 bed, public tertiary care hospital in Gilgit City that serves as regional medical center and caters to population not only from Gilgit as well as the surrounding areas.

Here we provide the first report on RSV infections from a tertiary care hospital in GB in infants and children under 2 years of age with acute respiratory tract infections (ARIs) during 2011-12 winter season. The epidemiological data and molecular characteristic of detected RSV genotypes were analyzed.

## Methods

### Collection of samples and epidemiological information

From December 2011 to March 2012, one hundred and five nasopharyngeal swabs were collected from children attending outpatient department with clinical diagnosis of ARIs (based on WHO definitions) either as Bronchiolitis or admitted to District Head Quarters (DHQ) Hospital Gilgit with pneumonia or both. The specimens were collected within 1-7 days of illness onset. Informed written consent was obtained from the parents of all children who provided specimens. The study protocol was approved by the ethics committee of DHQ hospital, Gilgit. Demographic and clinical information was collected from all patients. The samples were collected after informed and written consent from the children’s parents/ guardians.

The specimens were immediately placed at 4°C in tubes containing 1.5 ml cold viral transport medium. All naso-pharyngeal specimens were transported to the National Institute of Health, Islamabad for further testing and analysis. Upon receipt, all specimens were vortexed and centrifuged at 1,500x*g* for 10 min at 4°C, and the supernatants were stored at -80°C until processed.

### RNA extraction Real time PCR protocol for RSV detection

RNA was extracted directly from 140 µl of sample supernatants with an RNeasy Viral mini kit (Qiagen, Valencia CA, USA), according to the manufacturer’s instructions. The RNA was eluted in 50-60µl DNase and RNase-free water. 5µl of the extracted RNA was used as template in 25µl real time PCR mix. The reaction was performed on ABI-7500 using a panel of oligonucleotide primers and dual labeled hydrolysis (Taqman®) probes according to the CDC protocol for Non Influenza respiratory viruses (kindly shared by Drs. Erdmann and Peret from CDC). The assay tested each sample for RSV (for universal detection of human RSV). Human RNase-P gene served as an internal positive control for human nucleic acid. No template/negative controls (NTC) and positive template controls (PTC) for all primer/probe sets were included in each run. A specimen was considered presumptive positive for RSV if reaction curves crossed the positive threshold line within 40 cycles.

### RT-PCR for G protein

RSV-A and RSV-B specific oligonucleotide primers were used for subtype detection and sequencing of the G protein as summarized in Table 1. The expected size of the PCR products for RSV-A and RSV-B were 0.3kb & 0.8 kb respectively.

**Table 1:** RSV A & B oligonucleotide primers and probes used in this study.

**S. No.**	**Primer Name**	**Sequence (5'–3'**)	**Reference**
**1**	**Real-time RT-PCR for RSV**		
	RSV Forward	GGCAAATATGGAAACATACGTGAA	*US-CDC protocol (unpublished*)
	RSV Reverse	TCTTTTTCTAGGACATTGTAYTGAACAG	
	RSV probe	FAM-CTGTGTATGTGGAGCCTTCGTGAAGCT-BHQ-1	
**2**	**Conventional RT-PCR and Sequencing Primers for RSV-A**		
	***ExternalPCR***		*30*
	RSVA-G513-F	AGTGTTCAACTTTGTACCCTGC	
	RSVA-F131-R	CTGCACTGCATGTTGATTGAT	
	***NestedPCR***		
	RSVA-G606-F	AACCACCACCAAGCCCACAA	
	RSVA-F22-R	CAACTCCATTGTTATTTGCC	
**3**	**Conventional RT-PCR and Sequencing Primers for RSV-B**		
	Forward primer BGF	GCAGCCATAATATTCATCATCTCT	*31*
	Reverse primer BGR	TGCCCCAGRTTTAATTTCGTTC	
	BGF Seq.1	AGAGACCCAAAAACACYAGCCAA	
	BGR Seq.2	ACAGGGAACGAAGTTGAACACTTCA	

### Nested RT-PCR for RSV-A

In case of RSV-A, the second hypervariable region (HVR2) of the G-protein was amplified by nested PCR as described previously [29]. For cDNA synthesis, 12.5 µl of the total RNA was used as a template for reverse transcription-PCR (RT-PCR) with random hexamers for RSV-A. The external PCR was performed with 5 µl cDNA in a 50µl reaction mixture containing 25pmol of each of the primer pairs RS-AG513-F and RSVA-F131-R (Table 1), 100 µM for each dNTPs, 4 mM MgCl_2_, 0.5 U Platinum *Taq* DNA polymerase (Invitrogen), and PCR buffer (200 mM Tris-HCl, 500 mM KCl [pH 8.4]). Amplification was carried out at 94°C for 5 min, followed by 40 cycles of PCR, with 1 cycle consisting of 30 s at 94°C, 30 s at 58°C, and 1 min at 72°C, and a final extension step of 10 min at 72°C. The amplified products of 583 bp were analyzed by electrophoresis on a 1.5% agarose gel. In the case of negative results, 5 µl of the external PCR mixture was used for nested PCR, which was performed in a 50-µl reaction mixture with 25pmol of primers RSVA-G606-F and RSV-F22-R (Table 1). The cycling protocol was the same as for the external PCR, except for the annealing temperature, which was 53°C. The nested amplicons of 391 bp were visualized by agarose gel electrophoresis as well.

### RT-PCR for RSV-B

The reverse transcription (RT)-PCR assay was performed with a One Step RT-PCR kit (Qiagen) in a 50-µl volume containing 30 pmol each of forward and reverse primers and 10 µl of the extracted RNA (30). PCR reactions were conducted on a GeneAmp PCR system 9600 thermal cycler (Applied Biosystems, Foster City, Calif.) using following protocol; 55°C for 30 min for reverse transcription and 94°C for 15 min for DNA-polymerase activation; 40 cycles of 94°C for 30 s, 63°C for 1 min, and 72°C for 1 min, and final extension step at 72°C for 10 min. All the amplified products were subjected to 1.5% agarose gel electrophoresis. The amplicons were purified using a QIAquick PCR Purification kit (Qiagen).

### DNA sequencing

The PCR products were purified with a QIAquick PCR Purification kit (Qiagen), according to the manufacturer’s instructions. The purified PCR products were sequenced in the forward and the reverse directions on an ABI Prism 3130 genetic analyzer (Applied Bio systems) by using an ABI Prism BigDye Terminator cycle sequencing ready reaction kit 3.1 (Applied Biosystems) using sequencing primers. The sequence data obtained from this study has been deposited to GenBank under accession numbers KF437511 to KF437520.

### Phylogenetic Analysis

Three out of four RSV-B isolates and seven of RSV-A viruses from various age groups were included for sequencing and phylogenetic analyses. The nucleotide and deduced HVR2 amino acid (aa) sequences of Pak Gilgit (PG) RSV-A and -B were aligned with the RSV sequences retrieved from the GenBank database. For RSV-A, strain A2 (GenBank Accession No.X0221) was used as reference while CH18537 (Accession No. D00396) and prototype BA strain (BA4128/99B) were used for RSV-B.

Sequence data were compiled and edited using the Wisconsin Sequence Analysis Software Package version 10.0 (GCG, Madison, WI). Nucleotide and deduced amino acid sequences were aligned and phylogenetic trees for the HVR2 of G protein were generated using the neighbor-joining method with MEGA, version 5.05 [31]. Statistical significance of the tree topology was tested by bootstrapping in 1,000 pseudo-replicates while the evolutionary distances were derived using the Kimura-2 parameter method [32]. For both RSV groups A and B, phylogenetic trees were constructed by using reference sequences of representative strains from all known genotypes by using 240(RSV-A) and 300(RSV-B) nucleotides from the C-terminal variable region of the gene encoding G protein.

### Mutational analysis of deduced amino acids and N-glycosylation site analysis

Deduced amino acid sequences of the C-terminal second HVR of the HRSV G gene were generated by translating nucleotide sequences with the standard genetic code using MEGA software. These deduced amino acid sequences from HVR2 of G protein of both RSV-A and RSV-B strains were compared with prototype A2 strain and the RSV-B 18537 respectively for mutational changes. Potential N-glycosylation sites were predicted if the encoded amino acids were NXT, where X is not a proline [19]. For predication of O-glycosylation residues, the Net-O-Glyc server 3.1 was used [33].

### Statistical analysis of Epidemiological factors

Statistical analyses were performed with SPSS16.0 software. Significance of differences in frequencies of various demographic and clinical features and risk factors between various groups was tested using chi-square test and student’s t test. A *p* value of < 0.05 was considered to be statistically significant.

## Results

### Detection of HRSV and other viral agents

At least one respiratory virus was detected in 84 out of 105 samples (80%). RSV was the dominant virus and accounted for 71.4% (n=75) of the total cases. Out of these; 71 (94.6%) patients tested positive for RSV-A and 4 (3.8%) were positive for RSV-B. The remaining cases positive for viral respiratory infections included five with Influenza A, and four with Human Metapneumovirus (HMPV). Only four RSV-positive children were found to be co-infected with HMPV (6.25% of all positive cases).

### Epidemiology

**Figure 1:**
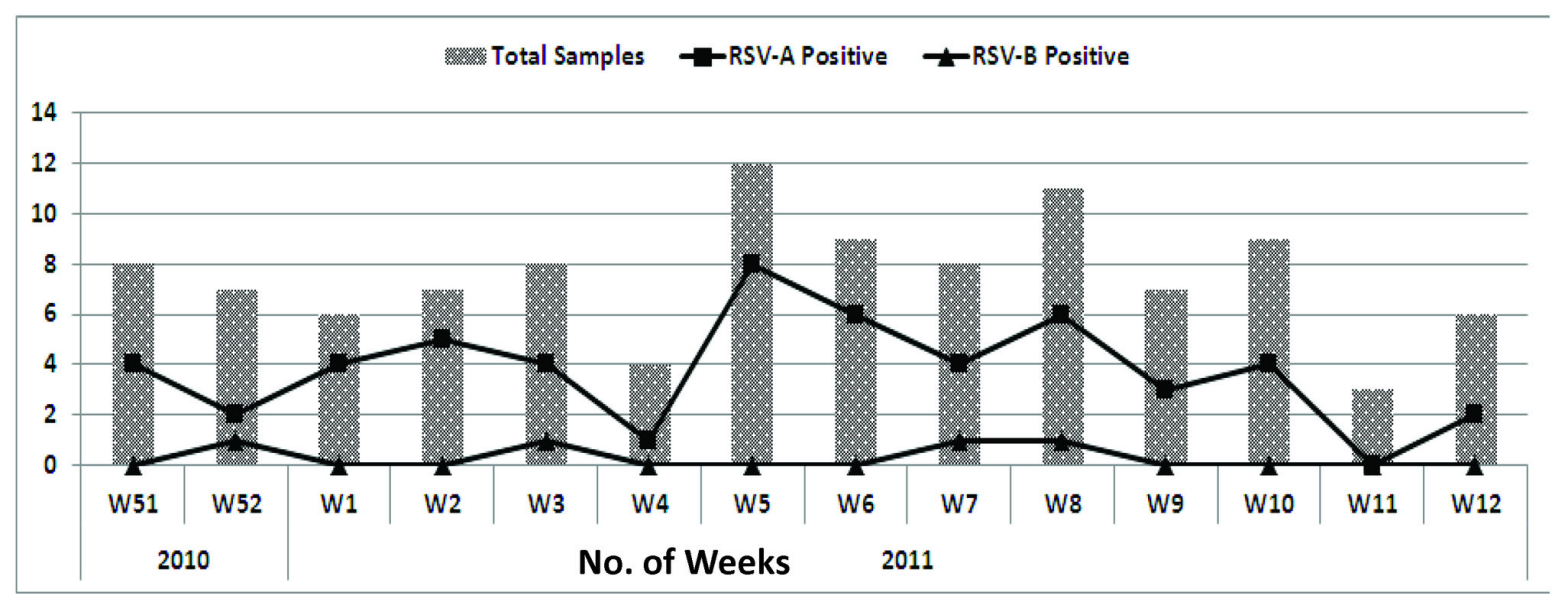
Weekly and Monthly Distribution of RSV-positive cases from Dec 2011-March 2012.

During the 2011-12 winter seasons, a total of 105 patients were enrolled who fulfilled the case definition of Acute Respiratory Tract infection (ARI) with 68% cases reporting in January and February (Figure 1). The gender ratio was 1.3:1 for boys to girls which was not statistically significant between RSV infected and RSV-negative groups (chi-square test; *p- 0.79*). and the ratio of outpatients to inpatients was 4.5:1. The mean age of RSV positive patients was 4.88 months+/- 2.5 and the age range was 26 days to 13 months. The age distribution of positive cases reflected the distribution for the received samples: 51% (n=positive/total: 42/54) of RSV positive cases were infants between 2.01 to 6 months age, 24% (19/25) were those aged 7 to 12 months followed by 16% (n=12/17) in 0-2 months and only 9% were between 1 to 2 years old. All tested patients were under two years of age (Table 2).

**Table 2:** Demographic details of RSV positive cases in children under 2 yrs.

**Age groups (months**)	**Total No. of Cases (%**)	**No. of RSV Positive Cases**	**No. of RSV Negative Cases**	***p-Value***
**Mean age ± SD**	105	4.88±2.2	4.26±2.23	*0.272*
0-2 months	17 (16)	12 (71)	5 (29)	*0.032*
>2-6 months	54 (51)	42 (78)	12 (22)	
>6-12 months	25 (24)	19 (76)	6 (24)	
>12-24 months	9 (9)	2 (22)	7 (78)	
**Gender**				
Male (%)	61 (58)	40 (73)	15 (27)	*0.28*
Female (%)	44 (47)	34 (53)	30 (47)	
**Mean Weight ± SD (Kg**)	105 (100)	6.64±1.80	6.45±1.84	*0.65*

### Clinical features of RSV infection

Information on clinical characteristics was available for all RSV positive patients and the most frequent clinical findings among them were cough (99%), fever (91.4%), and wheezing (90.5%). On uni-variate analysis of various clinical features such as cough, fever, crackles, wheezing, smoke exposure and presence of underlying conditions, no statistically significant differences were observed between RSV-positive and negative children (Table 3). Furthermore, positivity rates were similar for factors such as premature versus full term birth and home versus hospital delivery (*p*-values; 0.38 and 0.86). Bronchiolitis and Broncho-Pneumonia were the most frequent clinical diagnosis in RSV infected children (*p*-value: 0.15).

**Table 3:** Clinical and epidemiological characteristics between RSV positive and Negative cases.

**Birth History**	**RSV Positive n=75 (%**)	**RSV Negative n=30 (%**)	***p*-Value**
**Place of Delivery**			
Hospital	64 (71)	26 (29)	*0.86*
Home	11 (74)	4 (26)	
**Delivery**			
FTP	71 (72)	27 (28)	*0.386*
Premature	4 (57)	3 (43)	
**Clinical Manifestation**			
Fever (>38°C)	69 (72)	27 (28)	*0.741*
Cough	74 (71)	30 (29)	*0.525*
Wheezing	70 (72)	27 (28)	*0.229*
Difficult Breathing	55 (72)	21 (28)	*0.938*
Smoking Exposure	73 (79)	19 (21)	*0.172*
**Type of Patient**			
Outpatient	60 (70)	26 (30)	*0.423*
Inpatient	15 (79)	4 (21)	
**Final Diagnosis**			
Bronchiolitis	31 (76)	10 (28)	*0.152*
Broncho-Pneumonia	34 (64)	19 (36)	
Diagnosis unavailable	1 (9)	10 (91)	

### Phylogenetic and deduced Amino acid sequence analysis of G protein

**Figure 2:**
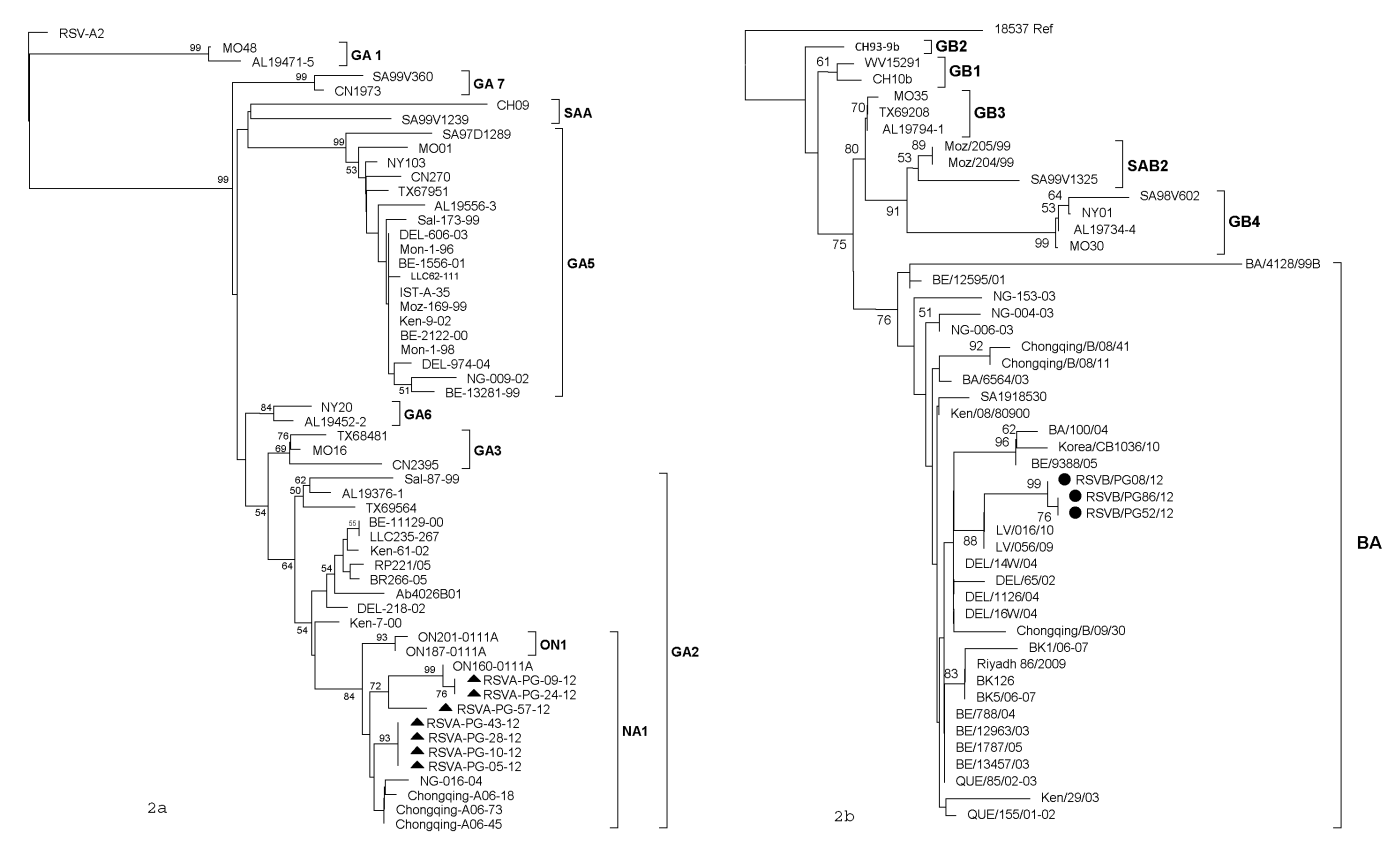
Phylogenetic tree of the second variable region of RSV group A & B genotypes and subtypes. The prototype strain A2 and 18537 were used as the out groups in the analysis. These trees were constructed using the neighbor-joining algorithm with 1,000 bootstrap replicates using MEGA 5. The genotypes are indicated by the brackets on the right side. Only bootstrap values over 50% are displayed at the branch nodes.

**Figure 3:**
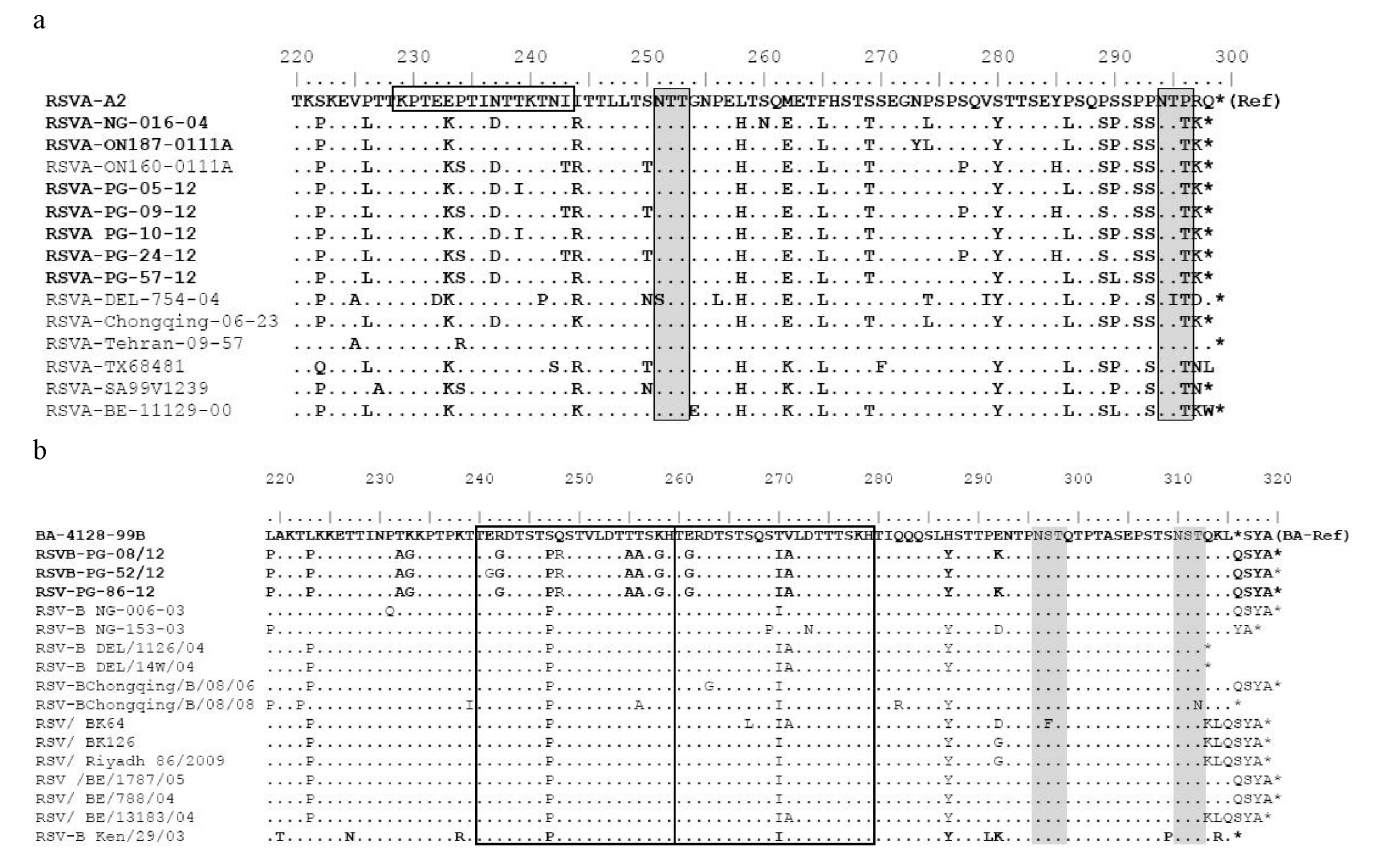
Deduced amino acid alignments of the second variable region of the G protein gene from RSV-A (a) and RSV-B (b) RSV-PG strains. Alignments are shown relative to the sequences of prototype strain A2 (GenBank accession number M11486) and genotype BA strain BA4128/99B (GenBank accession number AY333364). The amino acid numbering corresponds to strain A2 G protein positions 220 to 298 for the HRSV-A viruses and to strain BA4128/99B G protein positions 219 to 315 for the HRSV-B viruses. Identical residues are indicated by dots. Stop codons are indicated by asterisks. Potential N-glycosylation sites (NXT, where X is not a proline) are indicated by shaded blocks. In panel b, the two copies of the duplicated 20-amino-acid region in HRSV-B viruses are indicated by rectangles.

Out of 75 RSV strains detected in children with ARI, seventy one were subtyped as RSV-A and four as RSV-B respectively. Phylogenetic analysis of HVR2 nucleotide and amino acid sequences revealed that all analyzed RSV-A strains belonged to the GA2 genotype, clustering with sub-genotype NA1. The nucleotide and amino acid homologies among the strains ranged from 95.8 to 100% and 97 to 100%, respectively (Figure 2a). The rates of divergence between prototype strain A2 and the Pak Gilgit (PG) group A strains were 12.7% to 13.9% and 12% to 14% at the nucleotide and aa levels respectively. When compared with A2 strain, PG RSV-A G protein encoded 297 amino acids. On comparison to the reference A2 strain, specific amino acid substitutions for GA2 genotype were identified among PG RSV-A viruses including; S222P, P226L, E233K, N237D, I244R, L258H, M262E, F265L, S269T, S280Y, P283S, P286L, P289S, S290P/L, P292S, P293S, P296T, and R297K. PG RSV-A in viruses formed two sub-clusters; three viruses grouped with ON-160-0111 A strain (Canada) while others clustered with the Niigata (Japan) and Chongqing strains(China), with bootstrap values of > 70%. The P256L substitution was missing in Pakistani strains while at position 290, two samples retained the Serine residue; two strains had proline while only PAK GB/57/2012 had the classically reported S290L substitution (Figure 3a).

All Pak Gilgit RSV Subgroup B strains clustered into newly identified BA genotype (Figure 2b) The Pak Gilgit RSV B strains shared 91.8 to 100% nucleotide identity and 98.6 to 100% amino acid identity. There was 62% to 70% divergence at the nucleotide level and 25% to 29.2% divergence at the amino acid level between the PG group B strains and prototypic genotype B strain CH18537. The deduced amino acid sequences of all PG BA strains observed in this study had a predicted length of 319 aa due to duplication of 20 amino acids. Furthermore, in all PG RSV-B strains a six nucleotide deletion after residue 489 was also seen, resulting in a 2 amino acid deletion (sequence data not shown). Other amino acid mutations were observed at the following positions when compared to prototype strain; K218T, Q248R, T270I, V271A, H287Y (Figure 3b).

### N & O-glycosylation sites Analysis

Distinctive patterns of putative O- and N-glycosylation sites were seen among PG isolates. Only two putative N-glycosylation sites were identified on the second variable region of the G protein among the PG RSV-A strains. Both N glycosylated sites had NTT code at amino acid positions 251 and 294, and were conserved like prototype A2 and NA1/GA2 strains. The O-glycosylation profile was similar on analysis of 131 amino acids for PG RSV-A G protein and predicted up to 39 serine and threonine sites to be O-glycosylated with G scores of 0.5-0.7. Furthermore, among all the analyzed PG RSV-A strains, the reportedly conserved aa positions for serine at 267, 270, 275, 283, 287 and threonine at 227, 231, 235, 253, and 282 with high probability of O-glycosylation were preserved (Figure 3a).

Two N-glycosylation sites were identified among the group B strains (Figure 3b) at aa positions 296 and 310 at the C-terminal end of the G protein gene which are conserved among almost all the B strains. The O glycosylation site analysis of 254 amino acids predicted up to 64 potential sites for O-linked sugars attachment at serine and threonine residues in the ecto-domain of the HRSV-B G attachment protein(G scores of 0.5-0.7).

In addition to serine and threonine residues, the motif KPX*n*TTKX*n* (Figure 3a) was present in RSV-A protein sequences which is associated with extensive O glycosylation of the G protein This motif was not identified among the group B strains.

## Discussion

RSV represents a substantial burden of acute respiratory tract illness particularly in the early years of life leading to severe morbidity and hospitalization in very young children [9,16,26]. Knowledge of the RSV molecular epidemiology has been mainly based on studies done in developed countries [10,34,35]. Our findings show that RSV is an important viral respiratory pathogen contributing towards morbidity and mortality especially in very young children in Pakistan. In this study we analyzed G protein sequences of RSV-A and B isolates from clinical samples during winter 2011-12 in Gilgit Baltistan Province of Pakistan. Over 100 samples were collected from children with ARIs in a three month period and a significantly high rate of RSV infection (71.4%) was observed. RSV infections reached peak levels during mid-february and slowly tapered off towards end of March.

As DHQ hospital Gilgit is a tertiary care hospital that caters for a variety of patients, the samples that were collected for this study represent only a portion of the children with ARI. On the basis of an unusually high number of RSV positive cases from this isolated region, we deduce that this was in fact a seasonal outbreak of RSV. Other studies have similarly reported periods of intense/high RSV infections/epidemics during certain parts of the year [13,17,21,36,37]. Future surveillance of RSV prevalence and seasonality may help elucidate the disease burden for this region.

Previous studies suggest that male children are more susceptible to severe disease than females [12,19,21,35]. Similar results were obtained in our study and a higher percentage of RSV-infected children (55% n=39/71) were male, statistical analysis on clinical features and hospitalization rates between male and female patients did not reveal any striking differences. Age distribution analysis of our patients with RSV infection showed that infants younger than six months had the highest infection rates. Various studies have reported the highest incidence of RSV associated LRIs in developing countries in infants less than six months of age, and roughly two-thirds of RSV-LRI occur in children under two years of age (8,29). In our study, although the number of RSV infection in outpatients (n=60) was higher than inpatients(n=15), the RSV infection rates in outpatients (69.8%, 60/86) were lower than admitted cases (78.9%, 15/19). Therefore we reiterate that early testing for RSV in infants with ARI should be prioritized in order to ensure proper management. Furthermore, bronchiolitis and broncho-pneumonia were the most frequent clinical diagnoses in RSV positive cases from our data, as previously reported (12-14, 37,38), with RSV thought to account for approximately 85% of cases of bronchiolitis and approximately 20% of cases of childhood pneumonia.

The present study was implemented at only one hospital where children from adjoining as well as remote areas may consistently visit for years. Samples were collected mainly from children less than 2 years of age with acute upper and lower respiratory tract symptoms, such as wheezing, cough, rhinorrhea, and fever. It is well known that there is a reduction in the severity of clinical illness upon re-infection [14,15,38]. Since we do not have any previous data of RSV infections in the region nor could we obtain disease severity scores, we cannot discuss the differences in the clinical pictures with reference to any repeated infections. In addition, a limitation of the study is that there is a possibility of missing cases due to no visit to the clinic because of mild symptoms and considerable distances between villages and hospitals. Although information on hospitalization was available, the number of outpatients was high in comparison to admitted cases and no significant associations between subtype/genotype and disease severity could be established.

A higher number of RSV group A viruses were detected in comparison to group B strains in this study. Previous studies [4,12,15,28] have also documented a dominant global prevalence of RSV A subtype presumably on account of their higher level of genetic variability and divergence. The limited variability among the group B viruses might contribute to a more protracted spread of these viruses, leading to the predominance of group A over group B viruses in many studies of RSV epidemiology. Concurrently, it is also an established fact that various RSV subtypes and genotypes may co-circulate during one season and the predominant subtype may change from year to year [3,11,12,15,17,29,36,39]. On the basis of our limited data, we cannot make a comparison of the distribution pattern of RSVA and B subtypes at present but ongoing surveillance data will be able to better elucidate the prevalent pattern.

The HVR2 sequences of G protein of RSV-A viruses were only 94-97% homologous to sequences reported from neighboring countries including China and India and no identical sequences could be found in GenBank. This is understandable because limited sequence data is available at present time on the prevalence of RSV in SouthEast Asia and eastern Mediterranean region [10,40,51]. Conversely it is also known that viruses from different regions and various seasons apart may be more similar than those isolated from same area in one season [8,25,30,41-43].

The G protein of all analyzed RSV-A strains was found to be 297 amino acids in length, unlike the reference strain A2 which has a length of 298 aa. Other studies from neighboring India and China have also reported a predominance of GA2 subtype for RSV-A viruses with variable lengths of G protein [8,43,44]. Our data suggests that RSVA strains recently circulating in Gilgit region clustered with *NA1/GA2* genotype (bootstrap value of 92%) which originated in East Asia and spread globally [45]. Our RSV viruses are certainly closer to the earlier described Niigata *NA1* viruses from Japan as compared to the ON1 group of NA1 subtypes [12]. Furthermore, no novel ON1-specific substitutions were observed at E232G T253K, N273Y and P274L among the sequenced PG RSV-A strains (Figure 3a). However, due to the absence of genotyping data from past years in Pakistan, we are neither able to confirm any specific virus introduction/migratory patterns nor could we estimate the evolutionary rates for these isolates.

We report for the first time Pakistan group B strains that belong to the newly identified genotype BA, with a signature 60-nucleotide duplication in the HVR2 of the G protein first isolated in 1999 in Buenos Aires, Argentina [34]. The BA genotype was subsequently detected in Japan in 2003 [44] later in China [41], Thailand [37,46,47], South Africa [9], Brazil [52] and India [8], becoming the predominant HRSV-B genotype in circulation worldwide. All the group B strains carry the characteristic 60 nucleotide duplication, conserved stop codons, and a six nucleotide deletion in HVR1 [13,30,41,48]. Previous studies have reported variable G protein lengths for RSV B viruses isolated during the same season [5,30,41,49]. However, among the PG RSV B strains, the deduced amino acid homologies in HRV2 were 98 to 100% and only one G protein length of 319 aa was observed. Furthermore, the S247P amino acid substitution observed in the duplicated 20 amino acid region was also absent in the Gilgit RSV B viruses which puts them closer to the earlier described prototype BA strain. These RSV-B isolates also carry a six-nucleotide deletion after residue 489 (amino acid positions 159-160) in-frame deletion previously reported in Chinese strains from Chongqing and other studies [15,41]. Even though the number of RSV-B viruses analyzed in this study is too small, it is expected that future analysis of Pakistani strains will be helpful in understanding the significance of these mutations with a larger number of RSV-B isolates from consecutive seasons.

N- and O-linked glycosylation sites can influence the expression of certain epitopes by either masking or contributing to recognition by carbohydrate specific antibodies and aids viruses escape from the host immune response [50]. Since the G protein is highly glycosylated due to a high serine and threonine content, it is possible that the immunological impact of amino acid differences between strains is amplified by changes that occur at glycosylation sites [33]. Both PG group A and B strains had 2 N-glycosylation sites each within the second variable region of G protein (Figure 3). In case of the PG RSV-A viruses, the N-glycosylation sites reported at aa positions 237 & 250, a feature described in the NA1 sub-lineage of Japanese (Niigata) strains due to N237D substitution [44,45]. Furthermore, we have identified only nine Serine and Threonine residue positions to be potentially O glycosylated in the 60-nt duplicated region of RSV-B, while most studies report up to 10 potential sites (Figure 3b; position nos; 264 to 267, 269, 270, 274 to 276, and 280) [43]. It has been suggested that the modification of the number and location of the glycosylation sites may lead to continual circulation of certain genotypes, as the viruses evade neutralization by preexisting antibodies, This phenomenon is not unique to RSV alone and mutations in potential glycosylation sites in the haemagglutinin protein have been shown to provide a selective advantage for influenza viruses through production of more neutralization resistant virus progeny.

This is the first detailed report contributing critical preliminary data on the molecular epidemiology of RSV associated ARI in Pakistan. It highlights the significance of RSV as a dominant viral etiologic agent of pediatric ARI, and need for further work on incidence of viral pneumonias and their impact on public health. It is an established fact that various factors such as prevalent weather and climatic factors, status of pre-existing strain-specific immunity to the virus in the community would dictate which strains (present endemically in a community or introduced from outside) will predominate in successive seasons. Therefore, the importance of long-term molecular epidemiological surveys for early detection of prevalent strains and newly emerging genotypes cannot be over emphasized to foster better understanding of RSV infections in various regions of Pakistan.
