# Correction: Molecular Characterization of Circulating Respiratory Syncytial Virus (RSV) Genotypes in Gilgit Baltistan Province of Pakistan during 2011–2012 Winter Season

**DOI:** 10.1371/journal.pone.0145599

**Published:** 2015-12-17

**Authors:** Uzma Bashir Aamir, Muhammad Masroor Alam, Hajra Sadia, Syed Sohail Zahoor Zaidi, Birjees Mazher Kazi

An affiliation for the first author is not indicated. Uzma Bashir Aamir is also affiliated with #2: Atta-ur-Rehman School of Applied BioSciences (ASAB), National University of Science & Technology (NUST), Islamabad, Pakistan.
