# Correction: Correction: Molecular Characterization of Circulating Respiratory Syncytial Virus (RSV) Genotypes in Gilgit Baltistan Province of Pakistan during 2011–2012 Winter Season

**DOI:** 10.1371/journal.pone.0151009

**Published:** 2016-03-03

**Authors:** The PLOS ONE Staff

There is an error in the first author’s name in the article published on September 13, 2013 and in the correction published on December 17, 2015. The correct name is: Uzma Bashir.

The correct citation for the article is: Bashir U, Alam MM, Sadia H, Zaidi SSZ, Kazi BM (2013) Molecular Characterization of Circulating Respiratory Syncytial Virus (RSV) Genotypes in Gilgit Baltistan Province of Pakistan during 2011–2012 Winter Season. PLoS ONE 8(9): e74018. doi:10.1371/journal.pone.0074018.

The correct citation for the correction is: Bashir U, Alam MM, Sadia H, Zaidi SSZ, Kazi BM (2015) Correction: Molecular Characterization of Circulating Respiratory Syncytial Virus (RSV) Genotypes in Gilgit Baltistan Province of Pakistan during 2011–2012 Winter Season. PLoS ONE 10(12): e0145599. doi:10.1371/journal.pone.0145599. The publisher apologizes for the error.

## References

[1] Aamir UB, Alam MM, Sadia H, Zaidi SSZ, Kazi BM (2013) Molecular Characterization of Circulating Respiratory Syncytial Virus (RSV) Genotypes in Gilgit Baltistan Province of Pakistan during 2011–2012 Winter Season. PLoS ONE 8(9): e74018 doi:10.1371/journal.pone.0074018 24058513 10.1371/journal.pone.0074018 PMC3772930

[2] Aamir UB, Alam MM, Sadia H, Zaidi SSZ, Kazi BM (2015) Correction: Molecular Characterization of Circulating Respiratory Syncytial Virus (RSV) Genotypes in Gilgit Baltistan Province of Pakistan during 2011–2012 Winter Season. PLoS ONE 10(12): e0145599 doi:10.1371/journal.pone.0145599 26678561 10.1371/journal.pone.0145599 PMC4683100

